# Identification of artesunate as a specific activator of ferroptosis in pancreatic cancer cells

**DOI:** 10.18632/oncoscience.160

**Published:** 2015-05-02

**Authors:** Nils Eling, Lukas Reuter, John Hazin, Anne Hamacher-Brady, Nathan R. Brady

**Affiliations:** ^1^ Lysosomal Systems Biology, German Cancer Research Center (DKFZ), Heidelberg, Germany; ^2^ Systems Biology of Cell Death Mechanisms, German Cancer Research Center (DKFZ), Heidelberg, Germany; ^3^ Department of Surgery, Heidelberg University Hospital, Heidelberg, Germany; ^4^ BioQuant, University of Heidelberg, Germany

**Keywords:** artesunate, necroptosis, ferroptosis, KRas, pancreatic cancer, cell death

## Abstract

Oncogenic KRas reprograms pancreatic ductal adenocarcinoma (PDAC) cells to states which are highly resistant to apoptosis. Thus, a major preclinical goal is to identify effective strategies for killing PDAC cells. Artesunate (ART) is an anti-malarial that specifically induces programmed cell death in different cancer cell types, in a manner initiated by reactive oxygen species (ROS)-generation. In this study we demonstrate that ART specifically induced ROS- and lysosomal iron-dependent cell death in PDAC cell lines. Highest cytotoxicity was obtained in PDAC cell lines with constitutively-active KRas, and ART did not affect non-neoplastic human pancreatic ductal epithelial (HPDE) cells. We determined that ART did not induce apoptosis or necroptosis. Instead, ART induced ferroptosis, a recently described mode of ROS- and iron-dependent programmed necrosis which can be activated in Ras-transformed cells. Co-treatment with the ferroptosis inhibitor ferrostatin-1 blocked ART-induced lipid peroxidation and cell death, and increased long-term cell survival and proliferation. Importantly, analysis of PDAC patient mRNA expression indicates a dependency on antioxidant homeostasis and increased sensitivity to free intracellular iron, both of which correlate with Ras-driven sensitivity to ferroptosis. Overall, our findings suggest that ART activation of ferroptosis is an effective, novel pathway for killing PDAC cells.

## INTRODUCTION

Pancreatic ductal adenocarcinoma (PDAC) is an incurable form of cancer. Standard therapies increase survival rates by less than six months [1] and PDAC is predicted to increase in incidence [2]. PDAC is driven by constitutively-active KRas mutations [3, 4], which result in metabolic reprogramming [5-7] and resistance to apoptosis [8]. PDAC is highly resistant to death receptor and mitochondrial modes of apoptotic programmed cell death [8]. Moreover, death receptor activation is an important source of cancer-promoting inflammation signaling [9] and promotes metastasis [10]. Thus, the discovery of efficient strategies to kill pancreatic cancer cells remains an outstanding goal in programmed cell death (PCD) research, and considerable efforts are being made to identify general, as well as patient-specific, molecular targeting strategies [11, 12].

A complementary strategy, which we apply here, is to determine the mechanisms and mode of PCD activated by small molecules which induce efficient cancer cell death [13]. Here we focused on artesunate, a water- soluble derivative of the natural compound artemisinin, an effective anti-malarial [14], with well-understood pharmacokinetics [15, 16]. ART specifically induces cell death and blocks clonogenicity in a variety of cancer types [13, 17, 18, 19], including PDAC cells [20]. Importantly, ART-mediated cytotoxicity is dependent on increased reactive oxygen species (ROS) generation and the presence of iron [13, 21-23], and activates different modes of PCD, including apoptosis [13, 17, 21, 24], necroptosis [25], and lysosomal pro-death signaling [13, 19, 21].

Recent studies have revealed that oncogenic KRas mutations reprogram tumor cells to states dependent on enhanced glucose [7] and glutamine metabolism [6], which are required to support the upregulated antioxidant capacity needed for tumor growth [5]. Importantly, drug screening studies have recently uncovered that Ras transformation renders cells sensitive to a ROS-induced, non-apoptotic, iron-dependent mode of cell death [26, 27]. This mode of programmed necrosis, termed ferroptosis, is characterized by loss of redox homeostasis, increased lipid peroxidation, and inhibition by the small molecule ferrostatin-1 [28]. Intriguingly, PDAC redox homeostasis is dependent on cystine uptake via the ${\text{x}}_{c}^{-}$ transporter [29], which is a key participant in ferroptosis [30], suggesting an inherent sensitivity of PDAC to this iron-dependent mode of programmed necrosis.

Therefore, in the study presented here, we investigated the selectivity and mode of cell death activated by ART in PDAC cell lines. We report that ART induces an iron- and ROS-dependent cell killing and a block to clonogenicity in PDAC cell lines containing both wild-type and mutant KRas, but not control non-neoplastic HPDE cells. We report that co-treatment with either the ROS scavenger trolox, the inhibitor of ferroptosis, ferrostatin-1, or the iron chelator deferoxamine block ART cytotoxicity, while loading lysosomes with iron- saturated holo-transferrin enhances ferroptotic PDAC cell death. Moreover, our analysis of patient-derived mRNA expression data suggests that PDAC tumors *in vivo* can contain pathway adaptations that have been shown to sensitize Ras-transformed cells to ferroptosis. Overall, our findings suggest ART-mediated activation of the ferroptotic mode of necrotic cell death as a promising and highly effective pathway for killing PDAC cells.

## RESULTS

### ART induces iron-catalyzed, ROS-mediated PCD specifically in pancreatic cancer cells

**Figure 1:**
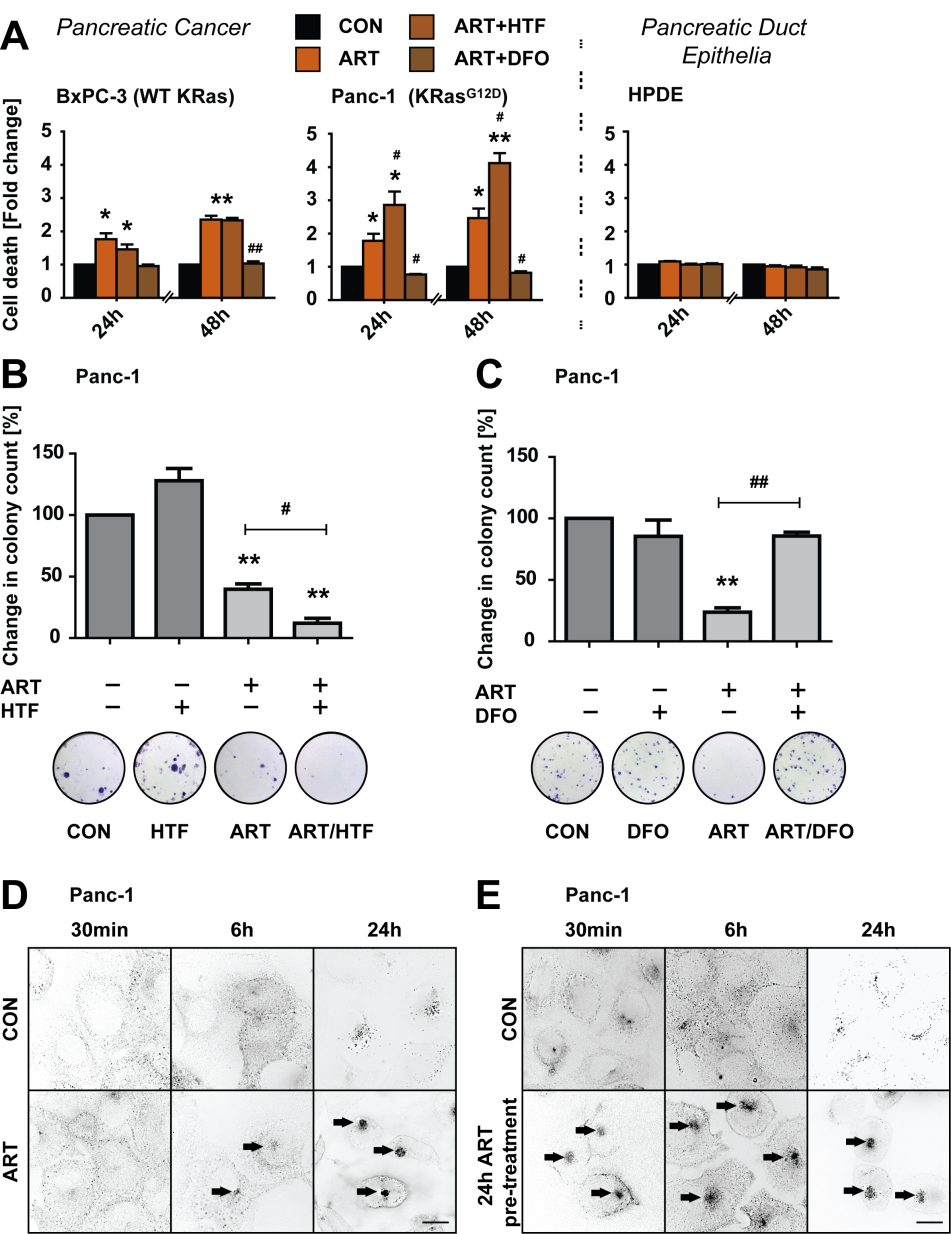
ART induces specific, iron-depended PCD in pancreatic cancer cell lines **A.** BxPC-3, and Panc-1 pancreatic cancer and non-neoplastic HPDE epithelial cells were treated with ART (50 μM) alone or in combination with iron-saturated holo-transferrin (HTF, 20 μg/ml) or the iron chelator deferoxamine (DFO, 0.1 mM) for 24 or 48 hours. Following, cell death was assessed using the exclusion dye PI (1 μg/ml). Data is presented as fold-change in PI intensity relative to drug-free control conditions. Statistical significance was tested *vs.* cells treated under control conditions (*) or ART alone (^#^)(*n* = 3; ^#^,*, *p* ≤ 0.05; **,^##^*p* ≤ 0.005). **B.** Panc-1 cells were subjected to ART, HTF, or ART and HTF. At 24 hours, 300 surviving cells were re-seeded for a colony formation assay. Colony count following 11 days of re-seeding is presented as fold change compared to control conditions. Statistical significance was tested *vs.* control (*) or ART alone (^#^) (*n* = 3-4; *,^#^, *p* ≤ 0.05; **,^##^, *p* ≤ 0.005). **C.** Panc-1 cells were subjected to ART, DFO, or ART and DFO for 24 hours. Following colony formation assays were performed and analyzed as in (B). **D.** Panc-1 cells were stained with Alexa Fluor Human Transferrin (HTF^546^, 5 μg/ml). Following, endolysosomal HTF^546^ was detected by fluorescence microscopy at 30 minutes, 6 hours and 24 hours fluorescence of exposure to ART or control conditions. Representative images of three independent experiments are shown. E, Panc-1 cells were pre-treated with ART or control conditions for 24 hours. Following, cells were stained with HTF^546^ and endolysosomal HTF^546^ fluorescence detected at 30 minutes, 6 and 24 hours. Representative images of three independent experiments are shown. Scale bars, 10 μm.

We first measured levels of ART-induced cell death at 24 and 48 hours of treatment in PDAC cell lines expressing wild-type KRas (BxPC-3) or constitutively active KRas^G12D^ (Panc-1) [31]. HPDE pancreatic duct epithelial cells [32] were used as a non-neoplastic control cell line to assess PDAC specificity of ART-induced PCD. PDAC cells were treated under nutrient deprivation conditions [13] to mimic the metabolic stress of PDAC [33, 34], while non-neoplastic HPDE cells were treated in fully supplemented medium. ART (50 μM) induced significant cell death at 24 hours in all PDAC cell lines, increasing at 48 hours (Figure 1A). Co-addition of the lysosomal iron chelator deferoxamine mesylate (DFO; 0.1 mM) [35] fully blocked cell death, demonstrating iron-dependency of ART-induced cell death in PDAC cells. Conversely, increasing lysosomal free iron by co- treatment with iron-saturated, diferric holo-transferrin (HTF; 20 μg/ml) significantly increased Panc-1 cell death at 24 and 48 hours of treatment. Control pancreatic duct epithelial HPDE cells were insensitive to all conditions, indicating tumor cell-specificity of death induction.

Next, to determine ART effects on long-term cell survival and proliferation we performed colony formation assays [36] following 24 hours of drug treatments. Consistent with cell death results, ART reduced clonogenic growth of Panc-1 cells, and this proliferative arrest was amplified by co-treatment with HTF (Figure 1B). Importantly, DFO rescued clonogenic growth inhibition induced by ART (Figure 1C), further highlighting a central role for lysosomal iron in ART-mediated effects.

HTF is endocytosed and trafficked to lysosomes [37], and we previously demonstrated that ART targets endolysosomes to the perinuclear region [13]. We therefore sought to determine if ART impacted HTF uptake in PDAC cells by measuring uptake of iron-loaded transferrin conjugated to Alexa Fluor 546 (HTF^546^, 5 μg/mL). In control cells, HTF^546^ entered cells and accumulated in endolysosomes distributed throughout the cytosol (Figure 1D). In cells treated with ART, HTF^546^ uptake was unaltered, as demonstrated by endolysosomal localization inside the cytosol. Moreover, within 6 hours HTF^546^-containing endolysosomes prominently formed clusters at perinuclear regions, consistent with previously demonstrated ART-induced perinuclear clustering of endolysosomes in breast cancer cells [13]. This perinuclear accumulation of HTF^546^ was also observed following 24 hour pre-treatment with ART, further demonstrating that ART treatment does not impair the lysosomal uptake of HTF (Figure 1E).

**Figure 2:**
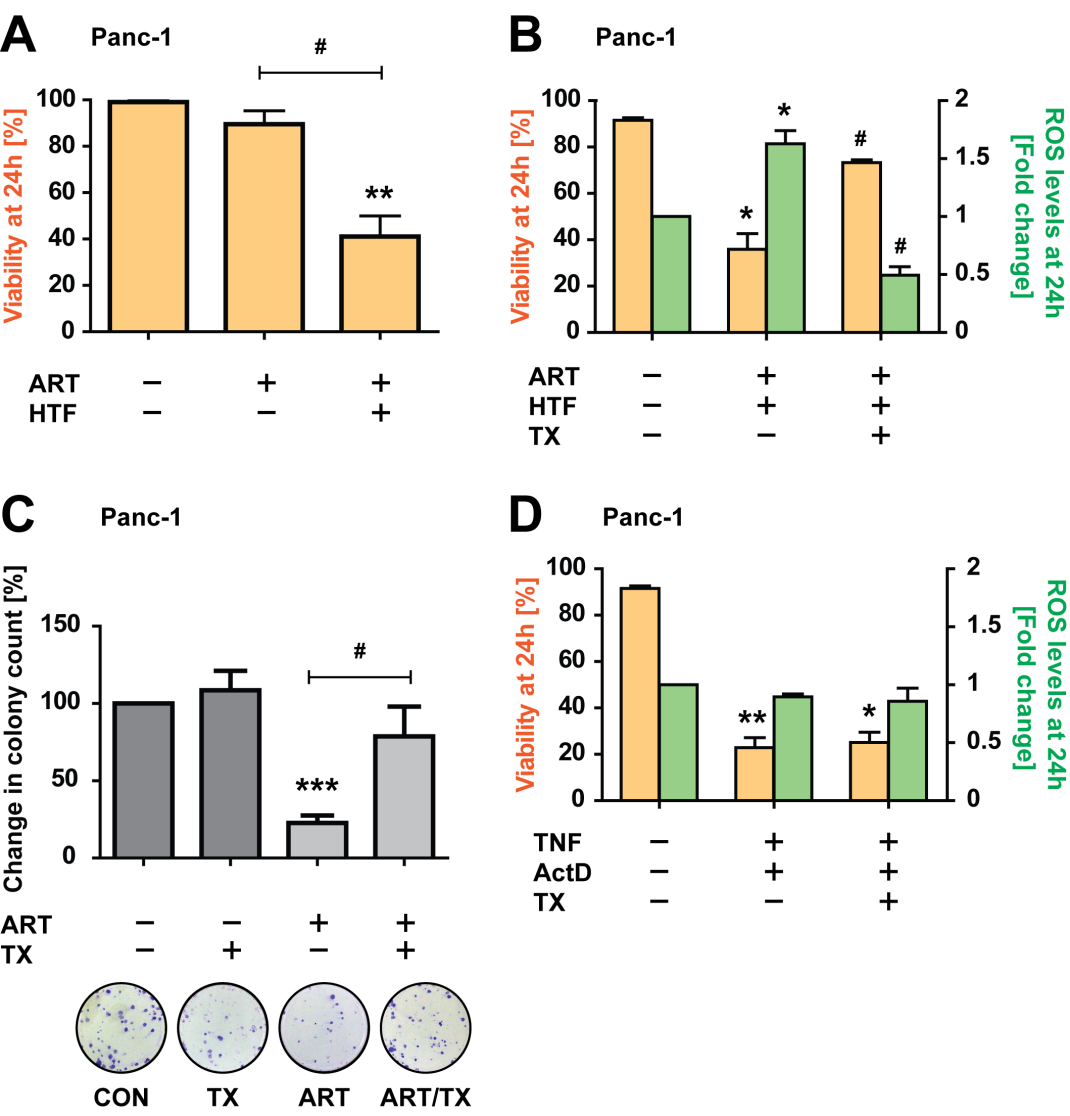
ART-induced, HTF-potentiated PDAC cell death is ROS-dependent **A.** Panc-1 cells were exposed to ART alone or in combination with HTF. At 24 hours cells were stained with PI and analyzed by imaging-coupled flow cytometry. The percentages of PI-negative cells are presented, as an index of viability. Statistical significance was tested *vs.* control (*) or ART alone (^#^) (*n* = 5; ^#^, *p* ≤ 0.05; **, *p* ≤ 0.005). **B.** Panc-1 cells were treated with ART and HTF, without and with addition of the antioxidant trolox (TX, 0.5 mM). At 24 hours, cell were stained with H_2_DCFDA and PI and analyzed by imaging-coupled flow cytometry. The percentage of PI-negative cells (viability) is shown (yellow bars). The mean DCF intensities of viable cells are presented normalized to control conditions (green bars). Statistical significance was tested *vs.* control (*) or ART alone (^#^) (*n* = 3; *,^#^, *p* ≤ 0.05). **C.** Colony formation assays were performed for cells exposed for 24 hours to ART or TX alone, or in combination. Colony count at 11 days post treatments is presented as fold change compared to control conditions. Statistical significance was tested *vs.* control (*) or ART alone (^#^) (*n* = 6; ^#^, *p* ≤ 0.05; ***, *p* ≤ 0.001). **D.** Panc-1 cells were exposed to TNF (43 ng/ml) and ActD (1 μg/ml), without and with TX. At 24 hours, cell were stained with H DCFDA and PI and analyzed by imaging-coupled flow cytometry. The percentage of PI negative cells (viability), and mean DCF intensities of viable cells, normalized to control conditions, are presented. Statistical significance was tested *vs.* control (*n* = 3; *,^#^, *p* ≤ 0.05; **, *p* ≤ 0.005).

Next, we determined whether PDAC cell death was dependent on ART-induced ROS generation [13, 17, 18], via imaging-coupled flow cytometry to measure ROS in parallel to cell death induction. At 24 hours ART/HTF treatment induced significant cell death (~60%), while ART alone did not induce significant levels of cell death (Figure 2A). Importantly, ROS generation by ART/HTF correlated with cell death, and the ROS scavenger trolox (TX; 0.5 mM) [38] significantly blocked cell death and reduced ROS levels (Figure 2B). Furthermore, TX rescued cell proliferative functions of ART-treated Panc-1 cells (Figure 2C). In contrast, treatment with TNF (43 ng/ml) and actinomycin D (ActD; 1 μg/ml), a combination known to induce apoptotic cell death in pancreatic cancer cells [39], resulted in TX-insensitive cell death (Figure 2D), demonstrating pathway specificity.

These results identify that ART specifically induces rapid ROS-mediated PDAC cell killing and long-term growth arrest, in a lysosomal iron-dependent manner.

### ART induces a MOMP- and caspase-3 independent mode of cell death

**Figure 3:**
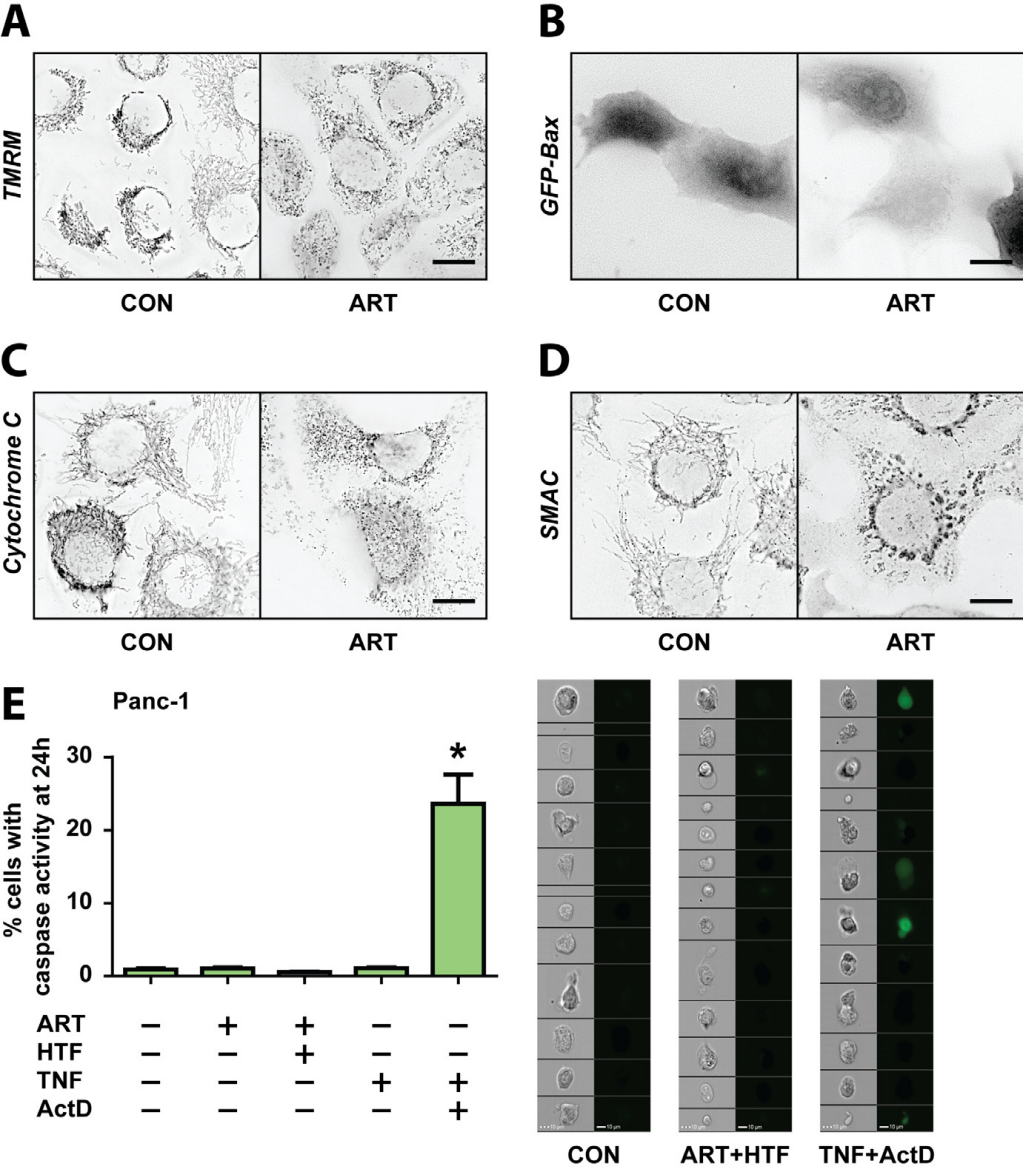
ART induces PDAC cell death in a non-apoptotic manner, independent of mitochondria- and caspase- mediated death signaling **A**.-**D.** Panc-1 cells were treated without and with ART for 24 hours. **A.** Cells were stained with TMRM following treatments and inspected by fluorescence microscopy. **B.** Representative images of cells that had been transfected with GFP-Bax prior to treatments. **C.** Cells were fixed and immunostained for cytochrome *c*. **D.** Cells were fixed and immunostained for Smac. Scale bars, 10 μm. **E.** Panc-1 cells stably expressing the fluorescent caspase-3 sensor, GC3AI, were treated without and with ART alone, ART and HTF, TNF (43 ng/ml), or TNF and ActD (1 μg/ml) for 24 hours. Following, GFP-positive, caspase-3 active cells were detected using imaging-coupled flow cytometry. The percentages of GFP-positive, caspase-3 active cells (left) and representative images of cells (right) are presented. Statistical significance was tested *vs.* control (*n* = 3; *, *p* ≤ 0.05).

We previously described that ART activated mitochondrial apoptosis in breast cancer cells [13]. We therefore examined different parameters of mitochondrial outer membrane permeabilization (MOMP), and downstream caspase activation in PDAC cells. Consistent with findings in breast cancer cells, ART induced mitochondrial fragmentation in Panc-1 cells at 24 hours of treatment (Figure 3A, 3C, 3D). However, Panc-1 cells maintained mitochondrial membrane potential under ART treatment, as shown by maintained TMRM fluorescence (Figure 3A). Furthermore, in Panc-1 cells ART did not induce mitochondrial translocation of GFP-Bax (Figure 3B), and did not trigger cytochrome *c* (Figure 3C) or Smac release (Figure 3D), all markers for MOMP activation [40]. Finally, use of a GFP-based caspase-3 activity sensor [41], which as expected reported TNF/ActD activation of caspase-3, demonstrated absence of caspase-3 activation by ART (Figure 3E). These findings demonstrate that, unlike breast cancer cells, Panc-1 cells resist ART- mediated activation of MOMP, and do not undergo neither caspase-dependent apoptosis, suggesting that in PDAC cells ART activates an alternative mode of PCD.

### ART does not activate necroptosis, but activates ferroptosis in PDAC cells

**Figure 4:**
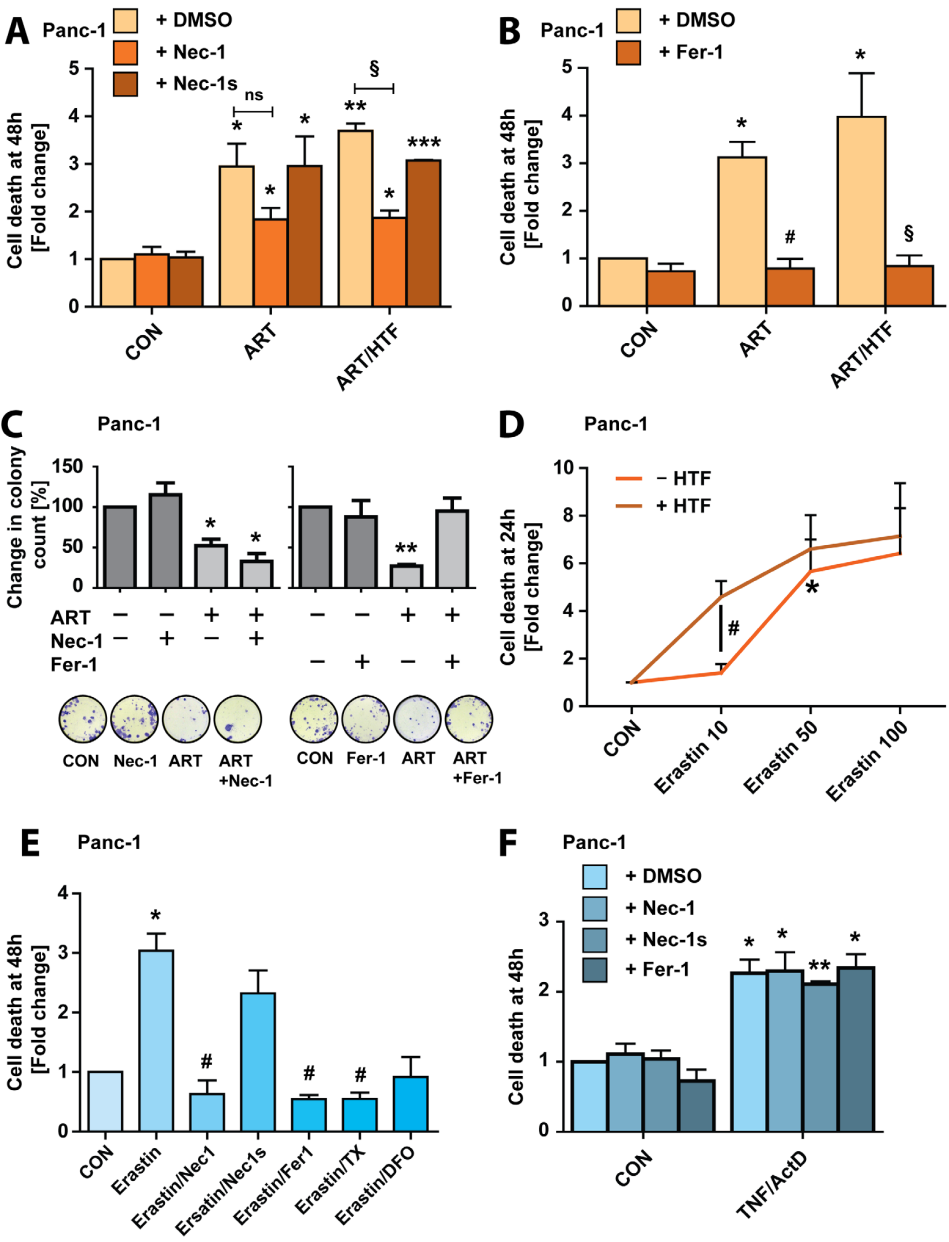
ART induces ferroptosis in Panc-1 cancer cells **A.** Panc-1 cells were subjected to ART or ART/HTF with and without addition of Nec-1 (20 μM) or Nec-1s (20 μM) for 48 hours. Following, cells were stained with Yo-Pro-1 and analyzed using a fluorescence plate reader. Cell death is presented as fold change increase in Yo-Pro-1 intensity normalized to DMSO control. Statistical significance was tested *vs.* control (*), ART (^#^), or ART/HTF treatment (§) (*n* = 3; *,^#^,§, *p* ≤ 0.05; **, *p* ≤ 0.005, ***, *p* ≤ 0.001). **B.** Panc-1 cells were treated with ART or ART/HTF without or with Fer-1 (20 μM) for 48 hours. Cell death was measured by Yo-Pro-1 fluorescence detection and is presented as fold change increase in Yo-Pro-1 intensity normalized to DMSO control. Statistical significance was tested *vs.* control (*), ART (^#^), or ART/HTF treatment (§) (*n* = 3; *,^#^,§, *p* ≤ 0.05). **C.** Colony formation assays were performed with Panc-1 cells subjected to Nec-1, Fer-1, or ART alone or ART in combination with Nec-1 or Fer-1. Colony count at 11 days post treatment is presented as fold change compared to control conditions. Statistical significance was tested *vs.* control (*) or ART treatment (^#^) (*n* = 3; *,^#^, *p* ≤ 0.05; ** *p* ≤ 0.005). **D.** Panc-1 cells were subjected to erastin (10-100 μM) without and with HTF for 24 hours. Cell death was measured by Yo-Pro-1 fluorescence detection and is presented as fold change increase in Yo-Pro-1 intensity normalized to control conditions. Statistical significance was tested *vs.* control (*), or 10 μM erastin (^#^) treatment (*n* = 3; *,^#^, *p* ≤ 0.05). **E.** Panc-1 cells were subjected to erastin (50 μM) alone or in combination with Nec-1, Nec-1s, Fer-1, TX, or DFO for 48 hours. Cell death was measured using Yo-Pro-1 and is presented as fold change in Yo-Pro-1 fluorescence intensity normalized to control conditions. Statistical significance was tested *vs.* control (*), or 50 μM erastin (^#^) treatment (*n* = 3; *,^#^, *p* ≤ 0.05). **F.** Panc-1 cells were subjected to TNF/ActD without or with Nec-1, Nec-1s, or Fer-1 for 48 hours. Cell death was measured with Yo-Pro-1 and is presented as fold change in Yo-Pro-1 fluorescence intensity normalized to control conditions. Statistical significance was tested *vs.* control treatment (*n* = 3; *, *p* ≤ 0.05; **, *p* ≤ 0.005).

ART was reported to activate necroptotic cell death in schwannoma cells [25]. We thus tested whether treatment with necrostatin-1 (Nec-1, 20 μM), a RIPK1 inhibitor [42], would reduce ART-induced PDAC cell death. Nec-1 reduced, but did not fully block, ART killing at 48 hours (Figure 4A). Therefore, we treated cells with the potent, more specific inhibitor of necroptosis, necrostatin-1s (Nec-1s, 20 μM) [43]. Nec-1s did not suppress cell death induction by ART (Figure 4A), suggesting that Nec-1 inhibition of ART-induced cell death was due to recently reported non-specific effects of the inhibitor [44], and evidencing that ART-induced Panc-1 cell death is non-necroptotic.

Interestingly, it was recently described that iron- dependent ROS production in Ras-transformed cells can activate programmed necrosis in the form of ferroptosis [28]. We therefore treated cells with the ferroptosis- inhibitor ferrostatin-1 (Fer-1, 20 μM), which resulted in full suppression of PDAC cell death at 48 hours of ART and ART/HTF treatment (Figure 4B). To further explore the induction of ferroptosis by ART, we performed colony formation assays with Panc-1 cells, following treatment with ART, ART/Nec-1 or ART/Fer-1. Nec-1 did not increase clonogenic growth under ART treatment, while Fer-1 rescued cells from ART-induced block to proliferation (Figure 4C), further suggesting ART activation of ferroptotic cell death. The small molecule erastin specifically induces ferroptosis by blocking the x - cystine/glutamate antiporter leading to glutathione depletion [28]. We thus analyzed the ability of erastin to induce ferroptosis in Panc-1 cells, at 10, 50 and 100 μM erastin concentrations, in absence and presence of HTF. Notably, at 24 hours 10 μM erastin induced significant cell death only with the addition of HTF. 50 μM erastin induced significant cell death alone, and was further increased by HTF co-treatment. 100 μM erastin induced maximal cell death, without further increase by HTF co-treatment (Figure 4D). We then characterized erastin (50 μM)-induced cell death in Panc-1 cells at 48 hours. Co-treatment with Fer-1, TX or DFO blocked cell death (Figure 4E). Importantly, Nec-1, but not Nec-1s, blocked erastin-induced Panc-1 cell death. These findings further indicate that Nec-1 exerts non-specific activity during ferroptosis, but not apoptosis, as neither Nec-1, Nec-1s nor Fer-1 blocked TNF/ActD induced cell death (Figure 4F).

**Figure 5:**
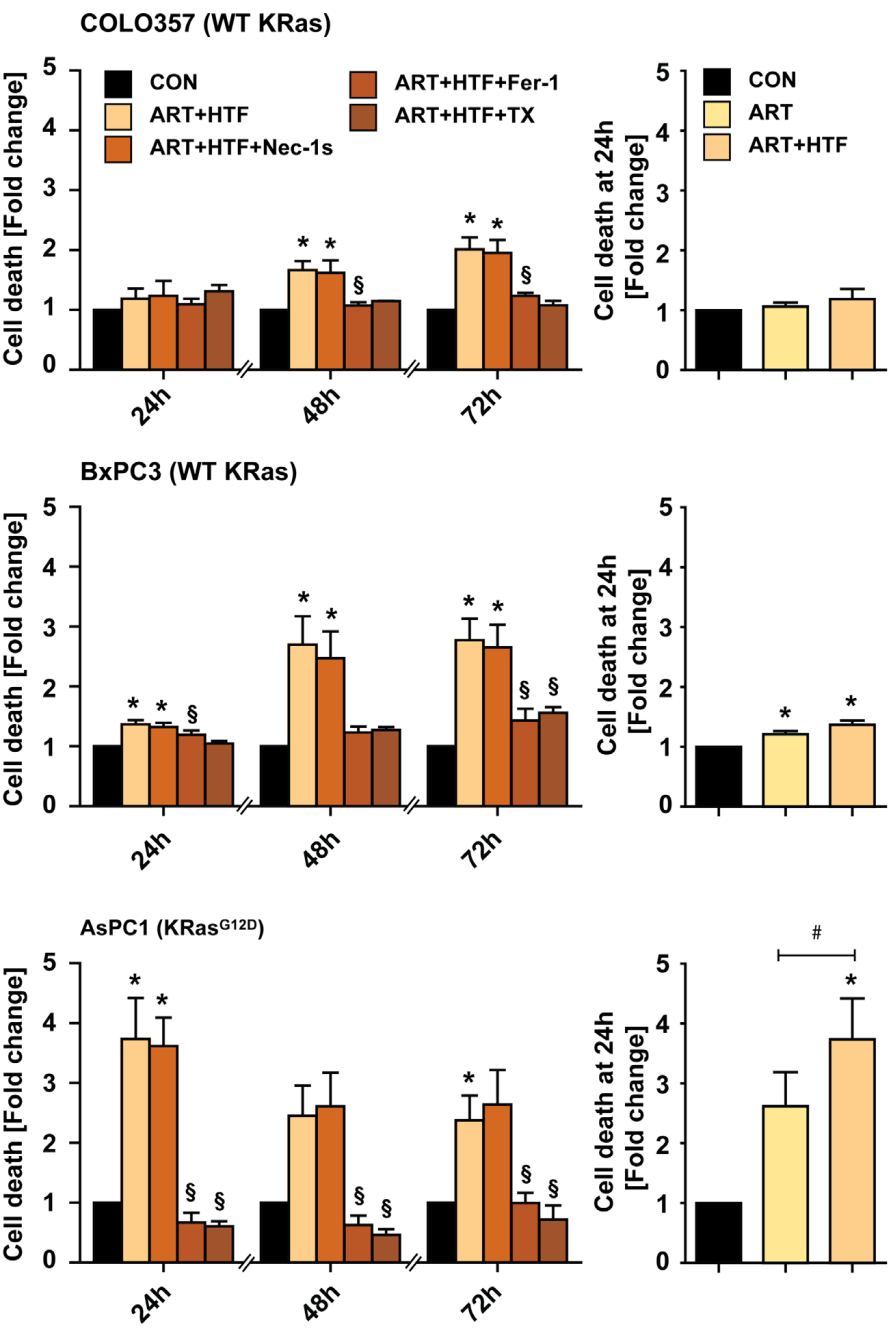
ART induces ferroptosis in a variety of pancreatic cancer cells COLO357 (WT KRas), BxPC-3 (WT KRas), and AsPC-1 (KRas^G12D^) PC cells were treated with ART/HTF without and with Nec-1s, Fer-1 or TX for 24, 48, or 72 hours (left panel of graphs). In parallel, cells were subjected to ART alone and compared to ART/HTF treated cells (right panel of graphs, cell death of ART/HTF is the same value as in left panel). Cell death was measured by Yo-Pro-1 fluorescence detection and is presented as fold change increase in Yo-Pro-1 intensity normalized to control conditions. Statistical significance was tested *vs.* control (*), ART (^#^), or ART and HTF treatment (§) (*n* = 3; *,^#^,§, *p* ≤ 0.05).

Ferroptosis was discovered as a pathway for killing cells transformed by mutationally-active Ras [26, 27]. Thus, we next compared HTF/ART-induced cell death and impact of Fer-1 in wild-type KRas (COLO357 and BxPC-3) and KRas^G12D^ mutant (AsPC-1) PDAC cell lines [31, 45] (Figure 5). COLO357 cells were overall less responsive to HTF/ART. Cell death was significantly induced in BxPC-3 (WT KRas) at 48 hours by HTF/ART and blocked by Fer-1. AsPC-1, which express KRas^G12D^ were most responsive to HTF/ART treatment, and Fer-1 blocked cell death fully, at all measured time-points. Notably, at 24 hours, ART alone induced significant cell death weakly in BxPC-3 and more potently in AsPC-1 cells. HTF enhanced killing only in KRas^G12D^ mutant AsPC-1 cells. The necroptosis inhibitor Nec-1s had no impact on cell death under any conditions.

Overall, these findings demonstrate that erastin and ART activate ferroptosis in PDAC cell lines in an iron- and ROS-dependent manner, and that ART-induced ferroptosis is most efficient in mutationally-active KRas expressing PDAC cell lines while Fer-1 block of cell death is independent from KRas mutation status.

### ROS signaling and lipid peroxidation during ART-induced ferroptosis

**Figure 6:**
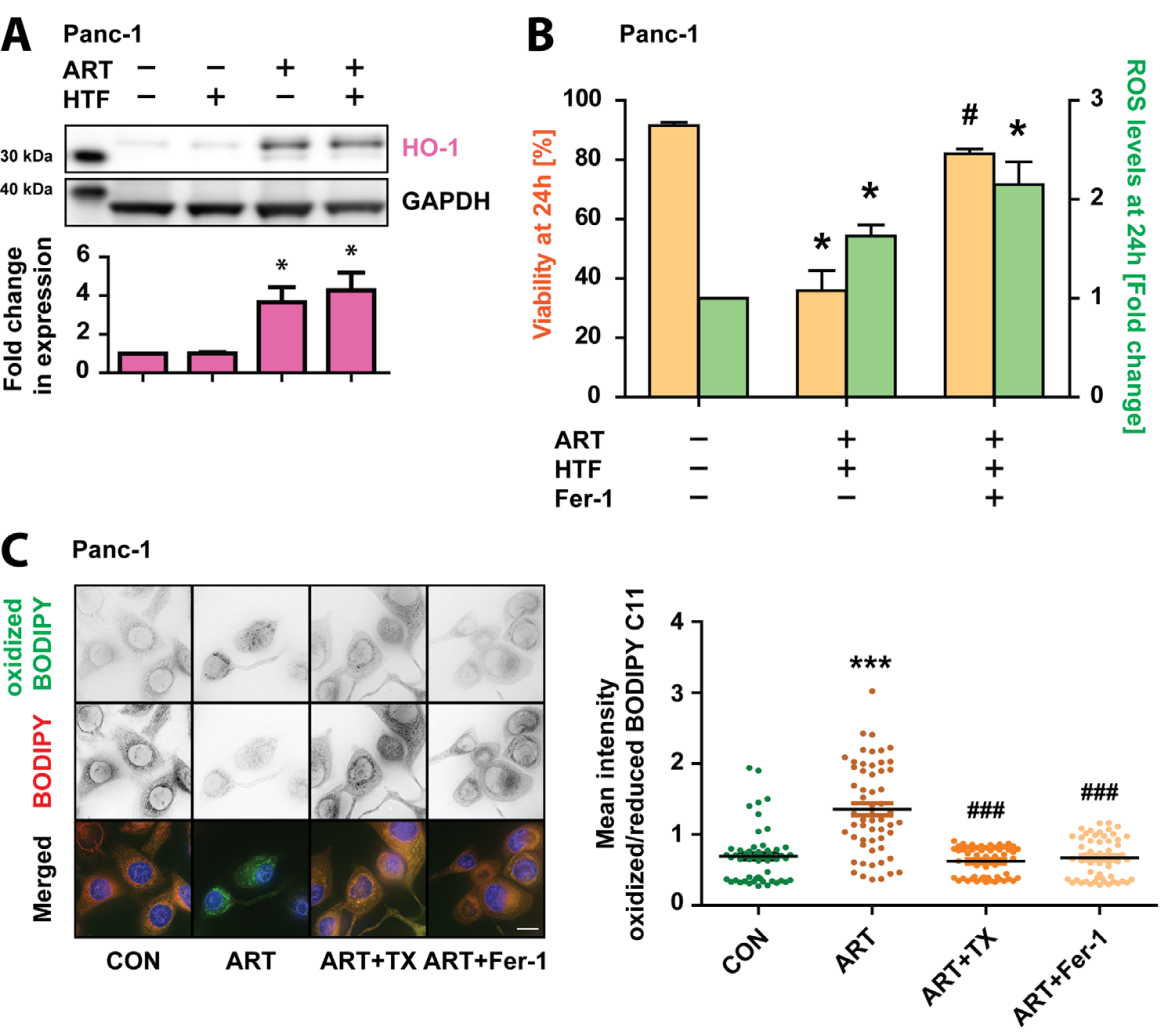
Ferroptosis in Panc-1 cells is characterized by lipid peroxidation **A.** Panc-1 cells were subjected to HTF, ART, or ART and HTF for 24 hours. Following, whole cell lysates were prepared and protein levels of Bach1 (92 kDa), and HO-1 (32 kDa) were detected via Western blotting. Graphs display quantified values, normalized to loading control GAPDH (37 kDa), and shown relative to control conditions. Statistical significance was tested vs. control conditions (*n* = 3; *, *p* ≤ 0.05, **, *p* ≤ 0.005). **B.** Panc-1 cells were treated with ART and HTF without and with Fer-1. At 24 hours, cells were stained with H DCFDA and PI prior to imaging-coupled flow cytometry. Percentage of PI negative cells (viability) as well as mean DCF intensity of viable cells normalized to control conditions are presented. Statistical significance was tested *vs.* control (*) or ART treatment (^#^) (*n* = 3; *,^#^, *p* ≤ 0.05). **C.** Panc-1 cells were seeded in 8-well microscopy μ-slides (iBidi) and exposed to ART alone or in combination with TX or Fer-1. At 24 hours, cells were stained with BODIPY C11 (581/591) and fluorescence of oxidized and reduced BODIPY C11 was detected. Lipid peroxidation is presented as single cell quantification of oxidized *vs.* reduced BODIPY C11 fluorescence intensity. 50 cells per condition were analyzed from three independent experiments. Statistical significance was tested *vs.* control (*), or ART (^#^) treatment (*n* = 3; ***, ^###^, *p* ≤ 0.001).

We next sought to characterize ferroptosis signaling in ART-treated Panc-1 cells. Iron-dependent ROS generation during ferroptosis is the central stressor for cellular damage and death [28]. Thus, we determined the impact of increased ROS by measuring the Nrf2-mediated antioxidant response [46]. Western blot analysis revealed that HO-1 expression increased in response to ART and ART/HTF (Figure 6A), indicating activation of ROS- mediated signaling pathways. To determine whether, similar to TX, Fer-1 blocks ferroptosis through ROS scavenging, we measured ROS generation in parallel to cell death in Panc-1 cells treated with ART/HTF without or with Fer-1. Surprisingly, imaging-coupled flow cytometry results indicated that Fer-1 blocked ART-induced loss of cell survival without blocking ART-induced ROS generation (Figure 6B).

We therefore asked if Fer-1 might act via blocking of localized ROS production in ART-treated Panc-1 cells, as Fer-1 was shown to inhibit lipid peroxidation during ferroptotic cell death [28, 47, 48]. Lipid peroxidation was measured using BODIPY C11 (581/591), a specific sensor for intracellular lipid peroxidation which undergoes a shift in fluorescence emission from red to green upon oxidization [49]. Panc-1 cells were treated for 24 hours with ART alone or in combination with TX or Fer-1 and then incubated with BODIPY C11 (581/591). Quantitative, single-cell analysis revealed a shift from non-oxidized to oxidized BODIPY C11 (581/591) in response to ART, evidencing increased lipid peroxidation (Figure 6C). Both TX and Fer-1 significantly reduced lipid peroxidation induced by ART.

### Characterization of PDAC *in vivo* potential for ferroptosis

While the above findings confirm that PDAC cell lines are insensitive to apoptosis, we here demonstrate that PDAC cells appear sensitized to ferroptotic cell death, induced by either ART/HTF or erastin treatments. We thus sought to determine whether cell culture-based understanding of factors contributing to apoptosis resistance and ferroptosis sensitivity are present in PDAC patient mRNA expression profiles. To that end, we analyzed the Badea dataset, which compares mRNA expression of 36 patient-matched tumor and normal pancreatic tissues samples [50].

### Iron and oxidative response

**Figure 7:**
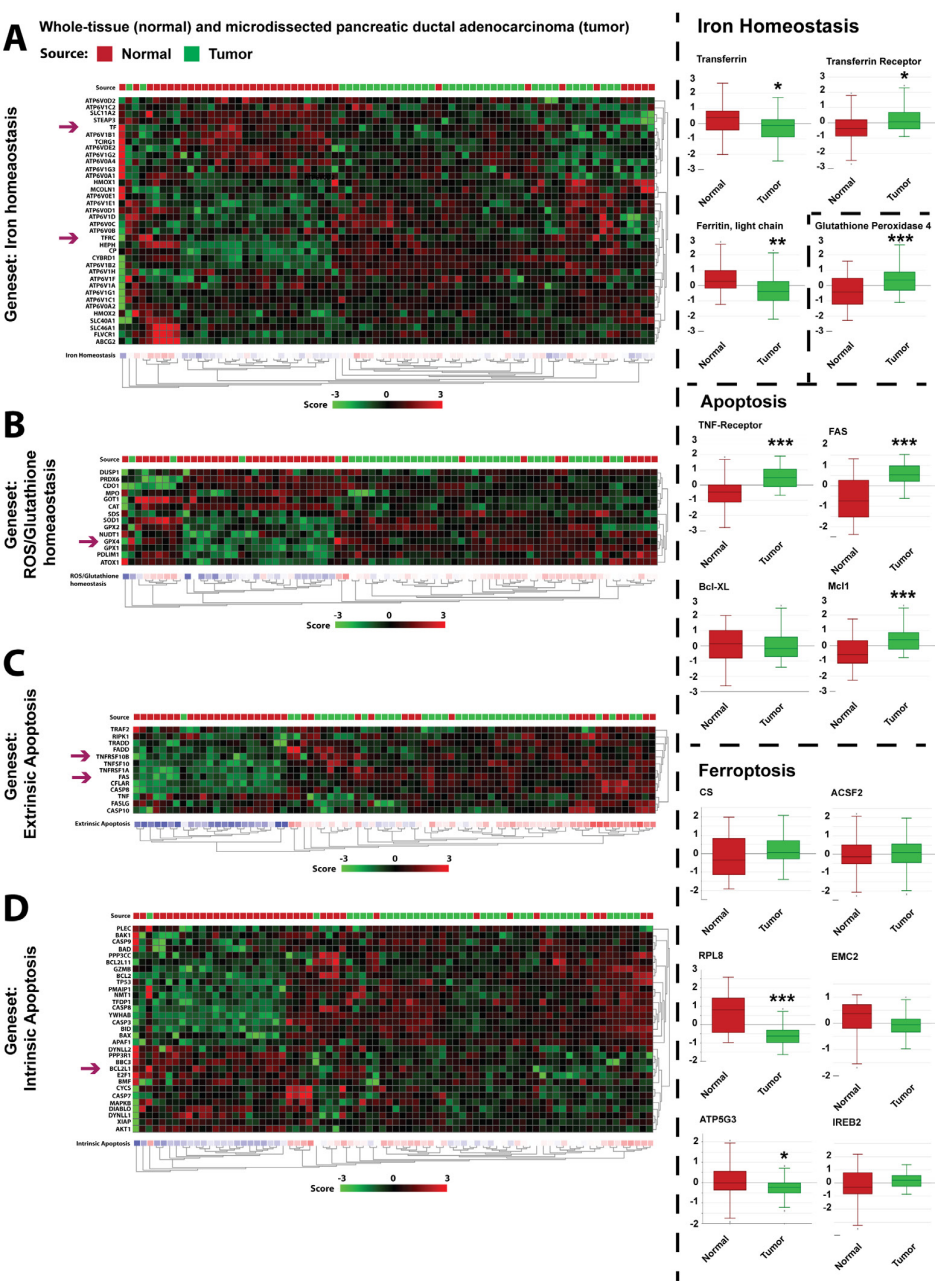
Analysis of patient gene expression data suggests PDAC *in vivo* sensitivity to ferroptotic signaling Gene set analysis was performed using R2 platform (r2.amc.nl) on the dataset from Badea *et al.* [50]. Tumor samples are indicated by green boxes while normal tissue samples are presented as red boxes. Differential expression is calculated based on the zscore showing a up-regulation (red) and down-regulation (green) clustered in heatmaps. **A.** Differential mRNA expression of genes contained in the iron homeostasis gene set. Differential single gene expression of transferrin (TF), TF-receptor (TFRC) and ferritin light chain (FTL) are presented as box plots. Statistical significance was calculated between normal and tumor tissue (ANOVA; *n* = 39; *, *p* ≤ 0.05; **, *p* ≤ 0.005). **B.** Differential mRNA expression of genes contained in the glutathione/ROS homeostasis gene set. Differential single gene expression of glutathione peroxidase (GPX4) is presented as box plots. Statistical significance was calculated between normal and tumor tissue (ANOVA; *n* = 39; ***, *p* ≤ 0.001). **C.** Differential mRNA expression of genes contained in the extrinsic apoptosis gene set. Differential single gene expression of FAS and TNF-Receptor (TNFR) are presented as box plots. Statistical significance was calculated between normal and tumor tissue (ANOVA; *n* = 39; ***, *p* ≤ 0.001). **D.** Differential mRNA expression of genes contained in the intrinsic apoptosis gene set. Differential single gene expression of Bcl-x_L_ (BCL2L1) are presented as box plots. Statistical significance was calculated between normal and tumor tissue (ANOVA; *n* = 39; ***, *p* ≤ 0.001). Additionally, box plots of expressed genes related to ferroptosis (CS, ACSF2, RPL8, EMC2, ATP5G3, and IREB2) are presented. Statistical significance was calculated between normal and tumor tissue (ANOVA; *n* = 39; *, *p* ≤ 0.05; ***, *p* ≤ 0.001).

Gene expression analysis revealed altered iron (Figure 7A) and ROS/glutathione (Figure 7B) homeostasis in patient PDAC tissues. In cell lines, Ras transformation results in increased levels of transferrin receptor (TFRC) and decreased levels of ferritin components [27]. TFRC, imports transferrin, and ferritin is responsible for the storage of intracellular iron, and serves as an important anti-oxidant [51]. Consistent with these findings, in the patient PDAC tissues, expression of TFRC is increased, while expression of transferrin (TF) and ferritin light chain (FTL) is decreased (Figure 7A). Glutathione peroxidase 4 (GPX4) is an inhibitor of ferroptosis that is dependent on GSH levels and high levels confer resistance to ferroptosis activation [48]. In the Badea dataset, the expression of GPX4, as well as other antioxidant enzymes are increased (Figure 7B), consistent with increased dependency on anti-oxidant system in PDAC [5].

**Figure 8:**
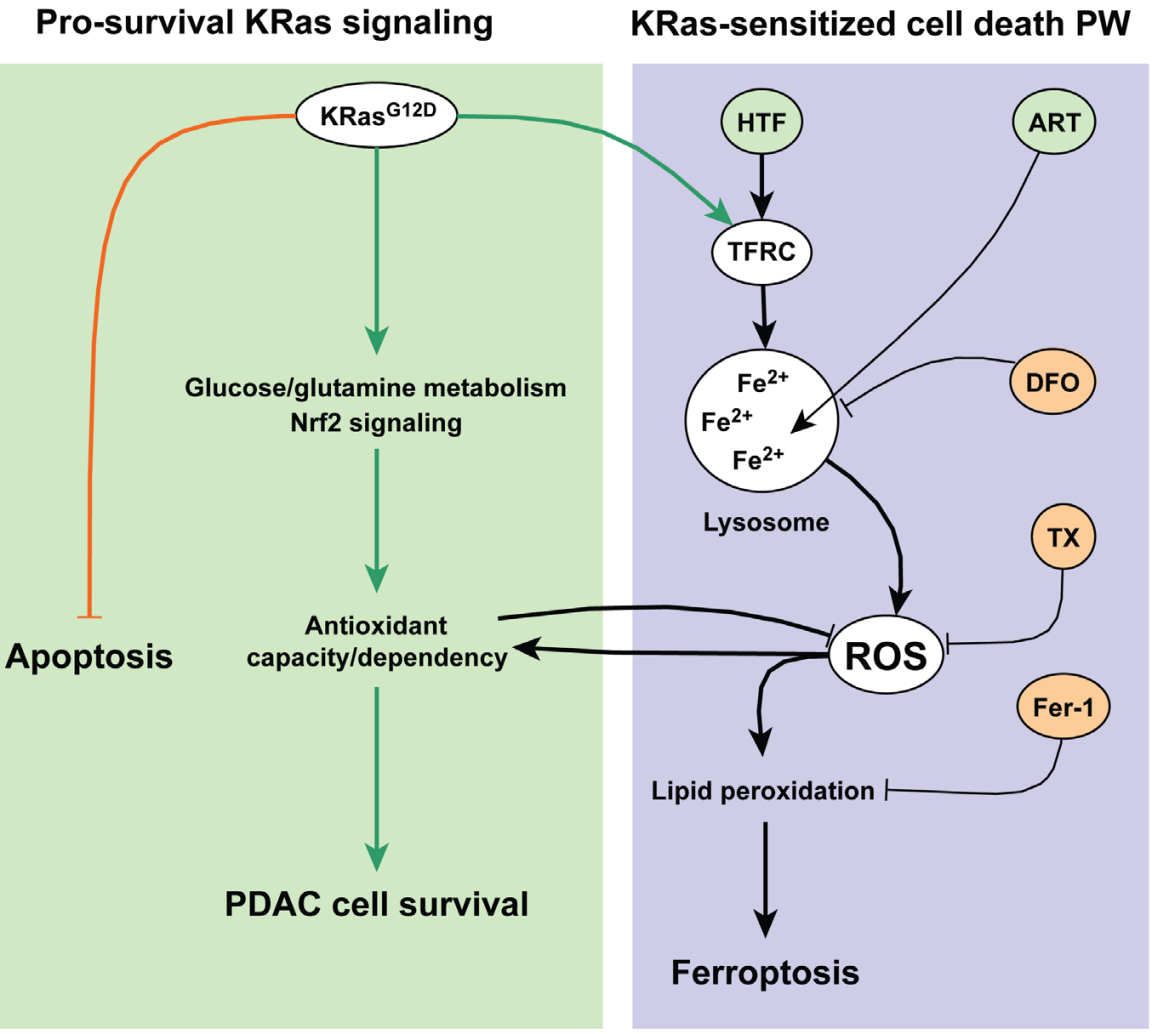
Schematic overview of ferroptosis induction by ART in pancreatic cancer cells PDAC cells carrying oncogenic Ras mutations undergo metabolic, antioxidant and anti-apoptotic adaptations, essential for cell survival. The antioxidant response counteracts toxic ROS and promotes cellular survival. ART interactions with lysosomal iron generates levels of ROS that overcome the capacity of the antioxidant response, leading to lipid peroxidation and ferroptotic cell death.

### Apoptosis resistance

Similarly, clustering apoptosis pathway gene sets of normal and tumor samples demonstrated a global apoptosis de-regulation in PDAC (Figure 7C-7D). Notably, TNFR and FAS death receptors are up-regulated in PDAC samples suggesting a pro-tumorigenic function (Figure 7C). This is consistent with the findings that TNFR expression promotes inflammation [9], and increased CD95 expression is responsible for PDAC metastasis [10]. Alterations in the mitochondrial pathway were also observed. For example, anti-apoptotic Bcl2 members MCL-1 expression was significantly increased, while Bcl-x_L_ had unchanged expression in PDAC patients (Figure 7D).

### Ferroptotic genes

Finally, we analyzed a set of six genes shown to be required for ferroptotic death [28], RPL8 (ribosomal protein L8), ATP5G3 (ATP synthase F0 complex subunit C3), IREB2 (iron response element binding protein 2), CS (citrate synthase), ACSF2 (acyl-CoA synthetase family member 2), and EMC2/TTC35 (ER Membrane Protein Complex Subunit 2). While IREB2, CS, ACSF2, and EMC2 showed no significant differences between normal and tumor tissues, RPL8 and ATP5G3 were significantly reduced in tumor tissues, due to patient heterogeneity. These findings suggest that patient profiling may be useful to suggest sensitivity to ferroptotic treatment strategies.

## DISCUSSION

Previously it was shown that ART activates ROS generation [13, 21-23], and lysosomal pro-death signaling [13, 19, 21], resulting in downstream activation of apoptosis [13, 17, 24] or necroptosis [25] pathways, in a cancer type-dependent manner. In this study we report that in PDAC cells, ART activates a form of cell death that is distinct from canonical, caspase-mediated apoptosis, and necroptosis. Instead, ART activates an iron- and ROS-dependent form of programmed necrosis, known as ferroptosis [28], with most effective ART-mediated death induction in PDAC cells expressing mutationally-active KRas.

The ferroptotic mode of programmed necrosis was recently discovered as an apoptosis-independent form of cell death in Ras-transformed cells [26, 27]. Ferroptosis is characterized by increased levels of lipid peroxidation, which can be caused by compound-mediated inhibition of the lipid peroxidase GPX4, via glutathione (GSH) depletion or through direct inhibition [48]. Here we show that the recently characterized ferroptosis-inducer erastin, which inhibits the cystine/glutamate antiporter system ${\text{x}}_{c}^{-}$ [30], activated ferroptosis also in Panc-1 cells, demonstrating the sensitivity of PDAC cells to ferroptotic cell death. This finding is consistent with the reported dependency of PDAC on system ${\text{x}}_{c}^{-}$ [29] in order to maintain redox homeostasis.

While mitochondrial apoptosis is critical for efficient chemotherapy induction of PCD in many cancer types [52], PDAC cells are highly resistant to apoptosis initiation and execution [4], and killing of PDAC cells represents an ongoing challenge in PCD research. Thus, the here described sensitivity of PDAC cancer cells to ferroptosis suggests an alternative pathway to selectively kill pancreatic cancer cells, especially considering that mutationally-active KRas mutations drive the majority of PDAC [7, 53, 54]. Importantly, we found that transferrin co-treatment increased ferroptotic cell death induced by both ART and erastin. Moreover, transferrin increased ART-mediated cell death to highest levels in Panc-1 and AsPC-1 PDAC cells, which have constitutively active KRas mutations [31], but did not alter the cell death response in BxPC-3 or COLO357 cells, which express wild type KRas [31, 45]. Indeed, iron-dependent killing of Ras-transformed cells offers a simple explanation for treatment specificity. Transferrin receptors are increased in PDAC patient tumor tissues [55, 56], and our analysis of the Badea dataset demonstrates TFRC up-regulation and down-regulation of ferritin in PDAC patients, recapitulating metabolic reprogramming by Ras which sensitizes transformed cells to ferroptosis [28]. Therefore, our cell culture findings suggest that, while PDAC cells are insensitive to apoptotic and necroptotic signaling, KRas mutation may render PDAC cells pre-sensitized to iron-dependent ROS-induction followed by ferroptotic cell death. Furthermore, our analysis of the Badea dataset not only indicates the high resistance of PDAC to apoptosis induction *in vivo*, but also a high degree of patient heterogeneity. It is possible, that certain patients may be more sensitized to induction of ferroptotic PDAC cell death.

At present, the only clinically-approved inducer of ferroptosis is the Raf kinase inhibitor sorafenib [57, 58], whose effectiveness in ferroptosis induction is limited by specific concentration ranges [30]. ART is well tolerated in malaria patients and pharmacokinetics have been characterized [15, 16]. Based on cell culture findings and patient-derived mRNA expression data, both indicating altered iron handling and antioxidant capacity in PDAC, we propose ART as a candidate for *in vivo* ferroptosis induction for targeted killing of PDAC cells.

## MATERIALS AND METHODS

### Reagents

Cell culture reagents were obtained from Invitrogen, Sigma, Lonza, and Pan Biotech. Electron microscopy-grade paraformaldehyde was obtained from EMS. Complete EDTA-free protease inhibitor and PhosSTOP phosphatase inhibitor cocktails were purchased from Roche. Alexa Fluor 546 Human Transferrin, tetramethylrhodamine methyl ester and 2′,7′-dichlorodihydrofluorescein diacetate were obtained from Invitrogen. Artesunate, holo-transferrin, ferrostatin-1, erastin and puromycin were purchased from Sigma. Trolox and actinomycin D were obtained from Calbiochem. Deferoxamine mesylate was purchased from EMD Bioscience. Necrostatin-1 was purchased from Santa Cruz Biotechnology. Necrostatin-1s was obtained from BioVision. TNF was a kind gift from BASF (Mannheim, Germany).

### Cell culture

Human pancreatic adenocarcinoma cell lines Panc-1, COLO357, AsPC-1 and BxPC-3 (obtained from the Department of General Surgery, University of Heidelberg, Germany), and the human embryonic kidney 293T cell line were maintained in DMEM supplemented with 10% FBS, L-glutamine, non-essential amino acids and penicillin/streptomycin/amphotericin B. The human pancreatic duct epithelial HPDE cell line [32] was maintained in KGM medium supplemented with bovine pituitary extract, hEGF, insulin, hydrocortisone, gentamicin and amphotericin B (Lonza). Fully supplemented media are referred to as full medium (FM).

### Drug treatments

Treatments of PDAC cell lines with artesunate (50 μM), holo-transferrin (20 μg/ml), erastin (10-100 μM), TNF (43 ng/ml) and actinomycin D (1 μg/ml), ferrostatin-1 (20 μM), necrostatin-1 (20 μM), necrostatin- 1s (20 μM), deferoxamine mesylate (0.1 mM), and trolox (0.5 mM) were performed in glucose-containing Hank's Balanced Salt Solution (in mM: 1.3 CaCl_2_, 0.5 MgCl_2_, 0.4 MgSO_4_, 5.3 KCl, 0.4 KH_2_PO_4_, 4.2 NaHCO_3_, 137.9 NaCl, 0.3 Na_2_HPO_4_, 5.6 D-glucose) to mimic the metabolically challenged conditions found in PDAC [33, 34]. Drug treatments of non-neoplastic HPDE cells were performed in full medium.

### Lentivirus-mediated gene transfer

For lentiviral gene transfer, a pCDH-puro-CMV lentiviral vector carrying the super folding (sf) GFP- tagged caspase-3 sensor (GC3A1), pCDH-CMV-GC3AI [41], was transfected into 293T cells together with pCMVdeltaR8.91 (packaging vector) and pMD2.G (VSV-G envelope protein expression vector) using calcium-phosphate transfection. Cells were infected with virus particle-containing supernatants at 50% confluency, and selected and maintained with puromycin (1 μg/mL). For experiments, cells were plated in puromycin-free medium.

### Colony formation assay

75,000 cells per well were seeded in 24-well plates and treated with the indicated drug combinations for a time period of 24 hours. Detached, dead cells were removed and after trypsinization, 300 cells per well were seeded into new 12-well plates and grown in full medium for 11 days to allow formation of colonies. Colony formation was inspected by widefield microscopy prior to fixation and staining with 1% (w/v) crystal violet in 25% MeOH for 20 minutes. Images were acquired using a Canon EOS 600D DSLR camera and colonies containing more than 50 cells were scored using the segmentation editor of Fiji software (http://fiji.sc/Fiji).

### High-resolution fluorescence microscopy

Widefield fluorescence microscopy was performed with a DeltaVision RT microscope system (Applied Precision) using a 60x objective. Cells were plated in 8-well microscopy μ-slides (iBidi), treated as indicated and subjected to either live-cell imaging or fixed with 4% paraformaldelhyde in PBS. For live-cell imaging, cells were stained with tetramethylrhodamine methyl ester (TMRM, 50 nM) for 20 minutes at 37°C or with BODIPY C11 (581/591) (0.5 mM) for 30 minutes at 37°C. Holo- transferrin uptake was monitored in live cells stained with Alexa Fluor 546 Human Transferrin (HTF^546^, 5 μg/ml) for the indicated amount of time. For immunofluorescence, fixed cells were permeabilized with 0.3% Triton-X in PBS, blocked with 3% BSA and incubated with antibodies against cytochrome *c* (BD Bioscience, no. 556432) or SMAC (Santa Cruz Biotechnology, no. sc- 22766) at room temperature for 2 hours. Fluorescent staining was performed for 1 hour at room temperature using highly cross-absorbed Alexa Fluor 488 or Alexa Fluor 546 secondary antibodies (Invitrogen). Images of representative cells were captured using the Z-axis scan function. Acquired images were deconvolved (Softworx, Applied Precision). Image analysis and preparation was performed using ImageJ (http://rsbweb.nih.gov/ij/) and Fiji (http://fiji.sc/Fiji). Representative images shown are total intensity projections of 5 μm thick Z-axis scans. For detection of lipid peroxidation, mean green intensity (oxidized BODIPY C11 (581/591)) was divided by mean red intensity (reduced BODIPY C11(581/591)) per cell.

### Imaging-coupled flow cytometry

To assess cell death in parallel to ROS levels, Panc-1 cells were treated as indicated, and incubated with propidium iodide (PI, 0.5 μg/ml) and 2′,7′-dichlorodihydrofluorescein diacetate (H DCFDA, 2 μM) for 1 hour at 37°C. Cells were then trypsinized and analyzed by imaging-coupled flow cytometry using the Imagestream X (Amnis). DCF and PI were simultaneously excited using the 488 nm and 546 nm lasers, respectively. Image analysis was performed using IDEAS 4.0 (Amnis). Briefly, single, in-focus cells were selected yielding 1000-2000 cells per condition for analysis. The percentage of PI-negative cells (survival) and the mean DCF values of PI-negative cells are reported.

### Fluorescence plate reader cell death assay

20,000 cells were seeded per well in 96-well plates and on the following day treated with the indicated drugs. At 24 and 48 hours, cells were stained with the cell exclusion dyes Yo-Pro-1 iodide 491/509 (0.1 μM) or propidium iodide (PI, 1 μg/ml) for 30 minutes at 37°C. Fluorescence read-out was performed using a Tecan Infinite M200 plate reader (Tecan) at 488 nm for Yo-Pro-1 stained cells or at 546 nm for PI stained cells. Cell death is presented as fold changes in Yo-Pro-1 or PI fluorescence intensity normalized to control conditions. To substract background, acquired intensities from non-stained wells were subtracted from stained wells prior to normalization:

$I=\frac{I_{Treat}-I_{Back}}{I_{Con}-I_{Back}}$, I_Treat_, (Intensity of stained, treated cells; I_Con_, Intensity of stained, control cells; I_Back_, Intensity of non-stained control cells).

### Western blotting

600,000 cells per well of a 6-well plate were plated and on the following day subjected to the indicated drug treatments. At 24 hours whole cell lysates were prepared using RIPA lysis buffer containing protease and phosphatase inhibitors. Dosed protein samples were electrophoresed using Bis-Tris NuPAGE gels (Invitrogen) and transferred to nitrocellulose using the iBlot dry blotting system (Invitrogen). Subsequently, membranes were blocked and incubated with antibodies against GAPDH (Santa Cruz Biotechnology, no. sc-25778) and HO-1 (Cell Signaling Technology, no. 5853S). HRP- conjugated secondary antibodies (GeneTex, GTX213110-01) were used for digital chemiluminescence detection. Blots shown are representative of three independent experiments. Densitometric band quantifications were performed using ImageJ (http://rsbweb.nih.gov/ij/). Briefly, the integrated intensities of target protein bands were measured and normalized to the integrated intensity of GAPDH loading control under the same condition.

### Statistical analysis

The probability of statistically significant increases or decreases between conditions of at least three independent experiments was determined using the Student's t-test. One-tailed paired t-tests were performed to test treatment versus control (*) while two-tailed, paired t-tests were performed to test co-treatment versus ART (^#^) or ART/HTF (§). Values are expressed for bar graphs and line graphs as mean ± SEM. Single cell data is presented as dot plot with mean ± SEM. Statistical significances and number of measurements are indicated in figure legends.

### Gene set analysis

Gene set analysis was performed using R2 platform (r2.amc.nl) on the dataset from Badea *et al.* [50]. Tumor samples are indicated by green boxes while normal tissue samples are presented as red boxes. Differential expression is calculated based on the zscore showing an up-regulation (red) and down-regulation (green) clustered in heatmaps.

## Abbreviations

ActD: Actinomycin D
ART: Artesunate
DFO: Deferoxamine
Fer-1: Ferrostatin-1
HTF: Holo- Transferrin
Nec-1: Necrostatin-1
Nec-1s: Necrostatin-1s
PDAC: pancreatic ductal adenocarcinoma
PCD: programmed cell death
TX: Trolox
TNF: Tumor Necrosis Factor
